# Cross-national validation of digital health engagement scales: evidence from 30 countries

**DOI:** 10.3389/fpubh.2026.1783814

**Published:** 2026-03-17

**Authors:** Petra Raudenská

**Affiliations:** Institute of Sociology, Academy of Sciences of the Czech Republic (ASCR), Prague, Czechia

**Keywords:** Bayesian methods, cross-national survey, digital health behavior, digital public health communication, ISSP, measurement invariance, online health information seeking, perceived usefulness

## Abstract

**Introduction:**

Reliable cross-national measurement of digital health engagement is essential for understanding how populations use online health information and for monitoring digital health inequalities. Yet standardized and psychometrically validated tools suitable for international surveys remain scarce.

**Methods:**

Using representative data from the International Social Survey Programme (ISSP) *Health and Health Care II module* (2021; *N* = 40,226; 30 countries), this study evaluated two newly developed instruments: (1) the *General Online Health Information Engagement* (GOHIE) scale, assessing the behavioral frequency and breadth of online health information seeking, and (2) the *Digital Health Usefulness* (DHUS) scale, capturing perceived usefulness of online health information. Reliability and construct validity were examined using item discrimination, nonlinear reliability estimation, and confirmatory factor analysis. Cross-national comparability was assessed using Bayesian Approximate Measurement Invariance (BAMI), which allows small probabilistic deviations in parameters across countries.

**Results:**

Both scales showed satisfactory internal consistency (*ω* = 0.70–0.90) and largely unidimensional structures in most countries. DHUS achieved stable approximate measurement invariance across national contexts, whereas GOHIE showed weaker item discrimination and greater contextual variability, particularly for the general information-seeking item and in countries with differing digital infrastructures or translation contexts.

**Conclusion:**

DHUS provides a robust cross-national measure of attitudinal aspects of digital health engagement, while GOHIE highlights the context-sensitive nature of behavioral online health information seeking and points to directions for future refinement. Cross-national analyses should examine country-specific parameters and apply approximate or partial invariance frameworks. These validated indicators establish a methodological foundation for monitoring digital health engagement and advancing equitable, evidence-based public health communication.

## Introduction

Accessing, understanding, and using health information are fundamental components of individual and population health. As health systems and everyday communication increasingly move online, the internet has become a dominant source for medical, preventive, and lifestyle-related information (1). This shift, accelerated by the COVID-19 pandemic (2), has transformed how people manage their health, interact with healthcare professionals, and participate in public health communication. Research on online health information seeking (OHIS) consistently shows that individuals use the internet not only to understand diseases and treatments but also to prepare for medical consultations, cope with uncertainty (3), and make informed decisions about prevention and self-care. Such engagement can enhance health literacy (4), self-efficacy (5), and preventive behavior (6), while also reshaping the dynamics of doctor–patient communication (7).

At the same time, digital health information environments entail substantial risks. The abundance of online content exposes users to misinformation, variable source quality, and cognitive overload (8). These challenges may reinforce existing social and digital inequalities: those with limited digital skills, lower education, lower trust in digital tools, or restricted access to technology are less likely to benefit from online health resources and more vulnerable to unreliable information (9). Consequently, digital health engagement has become an increasingly important indicator of public health equity and communication effectiveness.

In this study, OHIS refers specifically to the behavioral practice of searching for health-related information online. The broader term digital health engagement is used as an umbrella concept encompassing behavioral engagement (OHIS) as well as evaluative and attitudinal orientations toward digital health information, such as perceived usefulness.

Empirical research has documented relatively consistent sociodemographic patterns in OHIS. Women, younger adults, and individuals with higher education or income are more frequent users, whereas older adults and socioeconomically disadvantaged groups face barriers related to access, motivation, or digital competence (10). Beyond demographics, psychological and situational factors, such as health status, trust in health institutions, and the perceived reliability of online information, strongly shape engagement (11). These findings underscore that OHIS cannot be understood as a single, uniform behavior. Instead, theoretical frameworks such as the Comprehensive Model of Information Seeking (12), the Risk Information Seeking and Processing model (11), and the Planned Risk Information Seeking Model (13) conceptualize OHIS as a multidimensional phenomenon shaped by behavioral, attitudinal, cognitive, and contextual factors. These perspectives further emphasize that information seeking is driven by motivational and evaluative processes, including beliefs about the relevance and usefulness of information, as articulated in models of information seeking and technology acceptance (13–15). While these models have substantially advanced understanding of why and how people seek health information online, considerably less attention has been paid to how such multidimensional constructs are operationalized and measured in large-scale and cross-national survey research (16).

Despite rapid expansion of OHIS research, measurement approaches remain fragmented. Many empirical studies rely on single-item indicators or *ad hoc* survey questions that capture only selected aspects of information seeking and lack systematic psychometric validation. This weak alignment between theory and measurement limits replication and constrains meaningful comparisons across countries or cultural contexts (17). Existing validated instruments, such as eHealth literacy scales (18), primarily assess competencies rather than behavioral engagement or evaluative attitudes and are therefore not directly suited for monitoring OHIS in population-based surveys.

The International Social Survey Programme (ISSP), a long-standing cross-national survey initiative, responded to this gap in its Health and Health Care II module (2021) by introducing two new instruments designed to measure digital health engagement. The General Online Health Information Engagement (GOHIE) scale captures the behavioral dimension of OHIS by assessing the frequency and breadth of online health information seeking. The Digital Health Usefulness (DHUS) scale focuses on the attitudinal dimension, measuring perceptions of how useful online health information is for understanding, decision-making, and health management. Together, these instruments represent an important step toward more standardized measurement of digital health engagement in cross-national surveys.

However, these scales have not yet undergone systematic psychometric evaluation or measurement invariance testing. Given the central role of ISSP data in comparative social and public health research, the absence of psychometric validation for these scales poses a substantial risk for cumulative knowledge building and policy-relevant cross-national comparisons. Without such validation, it remains unclear whether they capture equivalent constructs across countries and whether observed differences reflect substantive variation rather than artifacts of translation, cultural norms, or response styles (19). Establishing reliability, construct validity, and measurement invariance of these indicators is thus a prerequisite for their use in comparative public health research.

This study directly addresses this gap by providing the first systematic cross-national validation of the GOHIE and DHUS scales using representative ISSP data from 30 countries (*N* = 40,226). Building on earlier conceptual and methodological work that highlighted the multidimensional nature of OHIS and the need for psychometrically sound measurement (16, 18), the present study extends this line of research to newly developed survey instruments. Specifically, it (a) assesses the internal consistency and construct validity of both scales; (b) examines their dimensional structure across national contexts; and (c) evaluates their cross-national comparability using Bayesian approximate measurement invariance (BAMI) testing.

By establishing whether these indicators function reliably and comparably across diverse populations, this study contributes both methodologically and substantively to research on digital public health communication. Methodologically, it demonstrates how advanced Bayesian invariance testing can be applied to newly introduced indicators in large-scale comparative surveys. Substantively, it provides validated tools for monitoring behavioral and attitudinal dimensions of digital health engagement, enabling more accurate assessment of digital inequalities and more robust cross-national comparisons. More broadly, this work strengthens the empirical foundations for integrating standardized indicators of digital health engagement into ongoing international monitoring systems and for informing evidence-based digital public health policy.

## Methods

### Study design and data source

This study used data from the ISSP Health and Health Care II module (fielded in 2021–2024). The ISSP is a long-term cross-national collaboration that conducts nationally representative surveys using standardized instruments and probabilistic sampling procedures, targeting individuals aged 16 years and older. The Health and Health Care II module includes items on health behavior, health care experiences, and, importantly for this study, new questions on digital health engagement.

Thirty countries participated in this module, covering Europe, the Americas, Asia, and Oceania. After excluding respondents without internet access (as the focal constructs require digital use), the analytical sample comprised 40,226 respondents (see Supplementary Table S1 for country-level details).

Each country selected the data collection mode most suitable for its research infrastructure: 15 countries used interviewer-administered modes (face-to-face, telephone, or computer-assisted interviews), while 15 applied self-administered modes (mail or online questionnaires). Because interviewer-administered modes may introduce additional sources of variability, through interviewer effects, paraphrasing, or respondent reactivity (20), data were analyzed separately for interviewer-administered and self-administered groups in the measurement invariance analyses.

The ISSP adheres to ethical research principles consistent with national regulations in each participating country. Data are fully anonymized and publicly accessible; therefore, additional ethical approval was not required for this secondary data analysis. Data and full documentation are available via the GESIS Data Archive (21).

### Measures

#### General online health information engagement (GOHIE)

The GOHIE scale measures behavioral engagement with online health information. It consists of four items assessing how often respondents used the internet during the past 12 months to seek: (1) general health or medical information, (2) information about healthy lifestyles, (3) information about stress, anxiety, or similar problems, and (4) information about vaccinations (see Table 1 for details). Response categories varied slightly across items (five- or six-point verbal frequency scales) and were reverse-coded so that higher scores indicate greater behavioral engagement.

**Table 1:** Overview of digital health engagement instruments used in the ISSP Health and Health Care II module (2021).

Scale (abbreviation)	Conceptual dimension	Item codes	Example item wording	Response categories
General Online Health Information Engagement (GOHIE)	Behavioral engagement – frequency and breadth of online health information seeking	v1–v4	**“D**uring the past 12 months, how often, if at all, did you use the internet on any device (e.g., computer, tablet, smartphone) to look for…? … **h**ealth or medical information for yourself or someone else / … **h**ealthy lifestyles / … **a**nxiety, stress, or similar problems / **… v**accinations”	v1: 1 = several times a day, 2 = once a day, 3 = several times a week, 4 = several times a month, 5 = several times a year, 6 = never or almost never.
				v2–v4: 1 = never, 2 = seldom, 3 = sometimes, 4 = often, 5 = very often.
Digital Health Usefulness Scale (DHUS)	Attitudinal evaluation – perceived usefulness of online health information and digital health tools	v5–v8	“**D**uring the past 12 months, information on the internet affected my health behavior in a positive way.” / “**D**uring the past 12 months, information on the internet helped me understand what a doctor tried to explain to me.” / “**T**he internet helps people decide if symptoms are serious enough to go to the doctor.” / **“T**he internet helps people check that doctors are offering appropriate advice.”	1 = strongly agree, 2 = agree, 3 = neither agree nor disagree, 4 = disagree, 5 = strongly disagree

Conceptually, GOHIE is understood as a composite indicator of behavioral engagement that brings together multiple online health information-seeking activities. Although these activities differ in content and situational relevance, they are assumed to share a common underlying orientation toward engaging with digital health information. Treating GOHIE within a latent variable framework therefore does not imply behavioral homogeneity, but rather serves as an empirical test of whether these heterogeneous activities can be meaningfully represented by a shared latent dimension. From this perspective, measurement invariance testing is a necessary analytical step to assess whether the assumed latent structure holds across national contexts and to delineate the limits of meaningful cross-national comparability.

#### Digital health usefulness (DHUS)

The DHUS scale captures attitudinal evaluations of online health information and digital health tools, reflecting perceived usefulness for understanding, decision-making, and health-related behavior (see Table 1). Responses were rated on a five-point agreement scale and reverse-coded so that higher scores indicate greater perceived usefulness.

While DHUS is theoretically rooted in the perceived usefulness construct of the Technology Acceptance Model (14) and related health communication theories (22), it extends beyond instrumental utility to capture a broader evaluative orientation toward digital health information. Specifically, the scale reflects beliefs about credibility, decision support, and personal agency in interactions with health information and healthcare providers, which are central to contemporary models of digital health engagement.

For cross-national analyses, items were standardized (*z*-scores) before estimation to account for differences in scale ranges and response distributions, as recommended for ordinal data in invariance testing (23).

#### Missing data treatment

The proportion of item nonresponse ranged between 0.1% and 7.6% for GOHIE and between 0.7% and 9.9% for DHUS items, with a few country-item combinations exceeding 10% (see Supplementary Table S1 for country-item details). Because listwise deletion could bias results and underrepresent countries with higher missingness, missing responses were handled within a Bayesian estimation framework that treats them as unknown parameters estimated within the model rather than removing them (24). This approach preserves full information and yields unbiased parameter estimates under the assumption of missing at random (MAR).

#### Analytical strategy

The validation process followed a multistep design integrating exploratory, confirmatory, and cross-national analyses.

1. Item diagnostics and reliability.

Item performance was first assessed using corrected item–total correlations (CITC) to examine each item’s discriminatory power within its scale. Items with CITC below 0.30 were considered weakly discriminating. Internal consistency was estimated using McDonald’s omega (*ω*) based on nonlinear SEM model with polychoric correlations, a reliability measure suitable for ordinal categorical data.

2. Dimensionality and construct validity.

To examine the underlying structure of each scale, Principal Component Analysis (PCA) was performed separately for each country. One-factor solutions with eigenvalues > 1 and factor loadings > 0.50 were interpreted as evidence of unidimensionality. Subsequently, Confirmatory Factor Analysis (CFA) tested whether the hypothesized single-factor model provided adequate fit in each national dataset. Model fit was evaluated using the Comparative Fit Index (CFI), Tucker–Lewis Index (TLI), Root Mean Square Error of Approximation (RMSEA), and Standardized Root Mean Square Residual (SRMR). Thresholds for acceptable fit were CFI/TLI ≥ 0.95 and RMSEA/SRMR ≤ 0.06. Conservative fit criteria were applied to avoid overestimating model adequacy in heterogeneous cross-national samples.

3. Cross-national measurement invariance.

To evaluate whether the scales measured equivalent constructs across countries, Bayesian Approximate Measurement Invariance (BAMI) was applied. Unlike traditional multi-group CFA, which imposes strict equality of parameters, BAMI introduces small-variance priors that allow for minor cross-group deviations in factor loadings and intercepts (25, 26). This probabilistic specification aligns with realistic assumptions about cultural heterogeneity in large international datasets and is more suitable for non-normal, ordinal data with heterogeneous sample sizes (27).

The analysis proceeded iteratively by increasing the prior variance (from 0.0001 to 0.05) until acceptable model fit was achieved. Fit was evaluated using Posterior Predictive Probability (PPP), 95% credible interval (CI), Bayesian RMSEA (BRMSEA), Bayesian CFI/TLI (BCFI/BTLI), and information criteria (BIC and DIC). Model fit was considered adequate when PPP > 0.05, 95% CIs including zero, BRMSEA ≤ 0.06, BCFI/BTLI ≥ 0.95, and improvements of ≥ 20 in BIC or ≥ 14 in DIC indicated better model performance. In practice, prior variances in the range of 0.01 to 0.05 proved suitable for large-scale cross-national surveys with heterogeneous samples, balancing flexibility in parameter estimation with overall model stability.

Countries that failed to reach configural invariance (i.e., diverging factor structure) were excluded from further BAMI steps, as cross-national comparability cannot be assumed without a common baseline model.

4. Software and reproducibility.

Descriptive analyses were conducted in IBM SPSS Statistics 29, while CFA and BAMI models were estimated in Mplus 8.11 using robust maximum likelihood (MLR) and Bayesian estimators. All analysis scripts and replication materials are available via the Open Science Framework (https://doi.org/10.17605/OSF.IO/JYFRB).

## Results

### Psychometric evaluation

Exploratory and confirmatory analyses assessed item performance, internal consistency, and factorial validity of the GOHIE and DHUS scales across all 30 countries (see Supplementary Tables S2, S3). Most items exhibited satisfactory discrimination, with corrected item–total correlations (CITC) exceeding 0.50, indicating strong coherence with the underlying construct. Several GOHIE items showed weaker discrimination (CITC = 0.20–0.29) in Austria, Mexico, Norway, Slovakia, Thailand, and the United States, reflecting greater contextual variability in behavioral engagement, while in Poland, item v1 (CITC = 0.005) displayed poor discrimination, suggesting potential translation or conceptual inconsistencies specific to that context. For the DHUS scale, weak discrimination across all items in India indicated limited coherence of the attitudinal construct in that context.

Reliability, estimated through nonlinear SEM methods suitable for ordinal data, was acceptable to excellent for most countries (*ω* = 0.70–0.90). Only India showed substantially lower reliability (*ω* ≈ 0.10), likely reflecting contextual or translation-related factors rather than data quality issues.

Principal component analyses (PCA) supported a predominantly unidimensional structure for both scales, with factor loadings typically between 0.60 and 0.80 and satisfactory Kaiser–Meyer–Olkin (KMO) criterion values (≥0.70). Weaker loadings and marginal KMO indices occurred in Denmark, Iceland, India, Mexico, Norway, Poland, Slovakia, Slovenia, Thailand, and the United States. The GOHIE scale produced a two-component solution in Poland, consistent with the poor performance of item v1. For the DHUS scale, local dependencies were observed between items v7 and v8 in several countries (e.g., Iceland, the Netherlands, Norway, and Slovakia), with inter-item correlations between 0.50 and 0.70, indicating partial conceptual overlap.

Single-group confirmatory factor analyses (CFA) confirmed that the hypothesized one-factor models fitted most national datasets adequately. For GOHIE, model fit was poor in India (CFI ≤ 0.90, RMSEA ≥ 0.10) and suboptimal in China, New Zealand, and Mexico (CFI ≤ 0.95, RMSEA ≥ 0.05), suggesting contextual differences in item interpretation. For DHUS, model fit improved consistently when correlating the residuals of items v7 and v8, confirming empirical redundancy between these indicators.

### Bayesian approximate measurement invariance (BAMI)

Measurement invariance of the two scales was evaluated using BAMI analysis, with interviewer-administered and self-administered data analyzed separately to account for potential mode effects (GOHIE-INT, GOHIE-SELF, DHUS-INT, DHUS-SELF).

For the GOHIE scale, full model convergence was not achieved for all countries, and based on posterior predictive checks, five samples (China, India, New Zealand, the Netherlands, and Switzerland) were excluded due to disrupted configural invariance, indicating differences in the underlying factor structure. Final models were selected based on convergence stability and Bayesian fit criteria (BRMSEA ≤ 0.06, BCFI/BTLI ≥ 0.95, ΔBIC ≥ 20 or ΔDIC ≥ 14). Acceptable model fit was obtained with prior variances of 0.05 for both GOHIE models and 0.05/0.025 for DHUS interviewer- and self-administered modes. Complete fit statistics are available in Supplementary Table S4.

Inspection of deviations in factor loadings and intercepts from the global mean identified the main sources of approximate non-invariance. Detailed item-level diagnostics and full heatmaps for both survey modes are provided in the Supplementary Table S5 and Supplementary Figures S1, S2. For GOHIE, metric invariance was largely supported, as most factor loadings showed only minor deviations across countries. Evidence for approximate scalar invariance was weaker: in interviewer-administered data, 52% of item–country intercepts significantly deviated from the global mean; while the proportion decreased to 44% in self-administered surveys. The strongest deviations were observed for item v1 (general online health information seeking), particularly in Thailand, South Africa, and the United States (18 of 26 countries in total), and for item v4 (vaccination information) in Slovakia and Israel (14 countries). These patterns may reflect heightened contextual salience during the COVID-19 pandemic. Item v3 (stress- and anxiety-related information) also showed moderate instability, particularly in Mexico, South Africa, and Russia, possibly capturing cultural variation in how emotional or coping-oriented information seeking is expressed. By contrast, item v2 (healthy lifestyle information) was the most stable across contexts.

Countries such as Taiwan, Hungary, Mexico, South Africa, Suriname, and the United States displayed consistent intercept deviations across several items, suggesting cultural or linguistic factors affecting response interpretation (28). Non-invariance in South Africa pointed to potential translation or conceptual issues. Despite these deviations, the GOHIE model achieved approximate scalar invariance according to Bayesian criteria, supporting conditional and context-dependent cross-national comparability.

Results for the DHUS scale were notably more robust. The interviewer-administered mode showed 33% significant intercept deviations, mainly for items v7 and v8 in Taiwan, Italy, and Poland. The self-administered mode performed best, with only 29% deviations and highly stable factor loadings across countries. Variability concentrated in v7–v8, which assess how individuals evaluate symptom seriousness and verify medical advice online, consistent with their higher inter-item correlations. Regional deviations were most pronounced in the Philippines, India, Italy, France, and Iceland, yet the overall structure remained stable and cross-nationally comparable.

Across all models, both scales reached approximate metric invariance, while the DHUS scale additionally approached scalar invariance, especially in self-administered mode. The residual non-invariance of GOHIE was localized to a limited number of items and countries, likely reflecting contextual differences in digital health information-seeking norms rather than measurement deficiencies (see Table 2).

**Table 2:** Summary of measurement invariance results.

Scale	Metric invariance	Scalar invariance	Most unstable items	Interpretation
GOHIE	Approx.	Partial	v1, v4	Context-sensitive behavioral indicator
DHUS	Approx.	Approx. (self-admin.)	v7, v8	Robust attitudinal construct

## Discussion

By systematically evaluating the psychometric properties and cross-national comparability of newly introduced ISSP indicators, this study advances comparative research on digital health engagement. The findings demonstrate how behavioral and attitudinal dimensions of online health information use can be measured in a conceptually grounded and empirically robust way across diverse national contexts. Combining classical psychometric diagnostics with BAMI testing, the study illustrates the conditions under which internationally comparable indicators of digital health engagement can be established in large-scale survey research.

From a psychometric standpoint, both scales demonstrated satisfactory internal consistency and factorial validity across diverse national samples. The Bayesian approach, permits small deviations from exact parameter equality, revealed that most cross-national differences in factor loadings and intercepts were minor and substantively negligible. However, differences emerged between scales and survey modes. Self-administered surveys were associated with higher levels of approximate invariance, reflected in lower parameter deviations and fewer non-invariant items. These patterns should be interpreted with caution, as survey modes were not randomly assigned across countries and may be confounded with contextual factors such as translation quality, fieldwork infrastructure, or national survey practices. Rather than establishing a causal mode effect, these findings highlight the importance of considering survey mode as a potential source of heterogeneity in cross-national research on digital health behavior.

The behavioral GOHIE scale showed greater cross-national variability and weaker inter-item coherence, reflecting heterogeneity in digital engagement norms and socio-technical environments. Items related to general information seeking (v1) and vaccination information (v4) exhibited the lowest discrimination and greatest instability, particularly in countries with differing digital infrastructures, health systems, or translation contexts. These differences highlight that OHIS is shaped by contextual salience, cultural norms, and public health events such as the COVID-19 pandemic. Although conceptually linked, the GOHIE items appear to capture distinct behavioral facets, preventive, lifestyle, or affective information seeking, whose relative importance varies across populations. From this perspective, partial non-invariance should be understood as informative about contextual differences in digital health practices rather than as a limitation of the indicator itself. At the same time, the item-level diagnostics reported here provide clear guidance for future refinement of the GOHIE scale, particularly with regard to items that are highly sensitive to contextual and temporal factors.

In contrast, the attitudinal DHUS scale demonstrated stronger psychometric robustness and higher cross-national comparability, indicating that perceptions of usefulness constitute a more stable and generalizable dimension of digital health engagement across national contexts. The factor structure remained stable across countries and modes, confirming a coherent attitudinal construct. Nevertheless, local dependencies between items v7 and v8, addressing the evaluation of symptoms and verification of doctors’ advice, suggest partial conceptual overlap. Their context sensitivity likely reflects variations in medical trust, healthcare accessibility, and digital literacy, all of which influence how individuals evaluate the reliability and relevance of online information.

Several methodological and conceptual limitations should be acknowledged. First, although BAMI provides a flexible approach to cross-national validation, it does not offer a fixed criterion for selecting prior variance. To ensure robustness, models with varying prior restrictions were tested following established recommendations (26); however, some diagnostic tools, such as the prior–posterior predictive *p*-value test, are not yet available for Mplus’ DIFF priors command (29). In addition, BAMI remains computationally demanding in large multi-group settings. Recent methodological advances, particularly Penalized Structural Equation Modeling (PSEM) (30) and machine-learning approaches (31), offer a promising avenue for addressing some of these limitations in future research.

Second, while internal consistency and factorial validity were rigorously examined, convergent validity was not systematically assessed for either scale. Future research should examine associations with theoretically related predictors, such as sociodemographic characteristics, digital and health literacy, trust in online information sources, and relevant health outcomes, to further establish external validity.

Third, this study relied on harmonized ISSP survey data without employing supplementary diagnostic approaches such as cross-national cognitive interviewing, web probing (32), or psychometric methods like Item Response Theory (33), which could help explain item-level differences across linguistic and cultural contexts. Addressing these limitations will further strengthen the precision and interpretability of cross-national indicators of digital health engagement.

### Implications for cross-national public health research

The findings highlight that different dimensions of digital health engagement require different analytical expectations in cross-national research. While attitudinal indicators such as DHUS show relatively high cross-national stability and are suitable for comparative analyses, behavioral indicators like GOHIE are more context-dependent and should be interpreted with greater sensitivity to cultural, infrastructural, and situational factors. Rather than assuming full scalar invariance, researchers are therefore encouraged to routinely assess approximate or partial invariance and to explicitly report the limits of cross-national comparability when analyzing digital health engagement in large-scale international surveys (34, 35).

Recommendations for future ISSP waves and international health surveys

1. Refine the behavioral scale structure and improve content validity. Future modules should clarify the conceptual scope of behavioral items, either consolidating them into a general engagement index or distinguishing subdimensions (e.g., preventive, affective, or lifestyle information seeking).
2. Strengthen linguistic and cultural equivalence in selected contexts. Additional pretesting, cognitive interviewing, and web probing may be particularly beneficial in countries where translation challenges or contextual differences are more likely to affect item functioning.
3. Integrate cognitive and attitudinal dimensions of digital health engagement. Including these indicators would allow researchers to disentangle behavioral engagement from structural and cognitive inequalities in digital health participation. Upcoming ISSP modules should also broaden or reframe OHIS measurement to include social media and digital health sources, which are increasingly central to information seeking, particularly among younger populations (36).
4. Standardize advanced invariance testing. Bayesian or alignment-based methods should become standard practice for longitudinal and cross-national validation of digital health engagement indicators.
5. Strengthen measurement through multi-item scales. To enable the assessment of measurement error and cross-national invariance, future international health surveys should prioritize standardized multi-item indicators over single-item measures whenever possible, rather than reducing online health engagement to simple frequency-based indicator.

## Conclusion

By systematically validating newly introduced ISSP indicators, this study strengthens the methodological basis for cross-national research on online health information seeking and perceived usefulness. The findings demonstrate that attitudinal evaluations of digital health information, as captured by the DHUS scale, are comparatively stable across national contexts, whereas behavioral engagement measured by GOHIE is more context-dependent and sensitive to cultural and infrastructural conditions. Rather than treating this variability as a limitation, the results clarify the scope and limits of cross-national comparability and provide guidance for the appropriate use of these indicators in international public health research.

## Data Availability

Publicly available datasets were analyzed in this study. The data: the data used in this study come from the International Social Survey Programme (ISSP) and are archived by [GESIS Data Archive] repository, [DOI: 10.4232/5.ZA8000.2.0.0]. These datasets are freely available for non-commercial research purposes after registration, ensuring compliance with ethical and data protection standards. All scripts, model specifications, and replication materials are available through the Open Science Framework (https://doi.org/10.17605/OSF.IO/JYFRB).
