# Supplementary material for: Cross-national validation of digital health engagement scales: evidence from 30 countries
Source: Front Public Health. 2026 Mar 17;14:1783814. doi: 10.3389/fpubh.2026.1783814 (PMC13036169; doi:10.3389/fpubh.2026.1783814)
Supplement: Supplementary file 1 [file Table_1.DOCX]

**Cross-National Validation of Digital Health Engagement Scales: Evidence from 30 Countries**

[anonymized]

**Supplementary Materials**

**Table S1** Number of respondents by country and missing values

| **Code** | **Country** | **N** | **Missing values in %** | | | | | | | |
| --- | --- | --- | --- | --- | --- | --- | --- | --- | --- | --- |
|  |  |  | **V1** | **V2** | **V3** | **V4** | **V5** | **V6** | **V7** | **V8** |
| AU* | Australia | 1010 | 4.7 | 5.0 | 5.3 | 5.2 | 9.3 | 9.7 | 6.6 | 7.3 |
| AT* | Austria | 1502 | 1.5 | 1.3 | 1.5 | 1.1 | 7.1 | 8.9 | 5.6 | 8.9 |
| CN | China | 1833 | 1.2 | 0.8 | 0.9 | 0.8 | 8.4 | **10.0** | 5.5 | 6.7 |
| TW | Taiwan | 1389 | 0.1 | 0.1 | 0.1 | 0.1 | 2.5 | 2.4 | 1.6 | 2.4 |
| HR | Croatia | 1068 | 0.3 | 0.9 | 0.7 | 0.7 | 6.3 | 8.4 | 3.2 | 4.1 |
| CZ | Czech Republic | 1148 | 0.3 | 0.7 | 0.9 | 0.7 | 6.2 | 6.9 | 2.3 | 2.6 |
| DK* | Denmark | 1666 | 2.0 | 2.5 | 2.9 | 1.9 | 6.9 | **10.3** | 8.9 | **11.9** |
| FI* | Finland | 982 | 1.4 | 0.8 | 0.9 | 0.8 | **11.0** | **17.4** | 6.2 | 7.5 |
| FR | France | 1584 | 0.6 | 0.9 | 0.8 | 0.8 | **11.9** | **15.4** | 2.7 | 2.0 |
| DE* | Germany | 1611 | 1.4 | 1.2 | 2.1 | 1.3 | 6.9 | 8.7 | 6.3 | 8.8 |
| HU | Hungary | 942 | 0.5 | 1.2 | 1.0 | 1.1 | **11.6** | **11.4** | **10.8** | **9.9** |
| IS* | Iceland | 1083 | 0.6 | 2.2 | 2.2 | 2.0 | 5.8 | 9.3 | 3.6 | 4.4 |
| IN | India | 1533 | 4.7 | 4.3 | 5.3 | 6.5 | **27.1** | **26.2** | **11.0** | **17.4** |
| IL* | Israel | 1183 | 0.3 | 0.2 | 0.3 | 0.5 | 6.2 | 8.0 | 1.0 | 1.4 |
| IT | Italy | 1061 | 0.8 | 0.7 | 0.6 | 0.7 | 9.8 | 8.7 | 7.2 | 7.8 |
| JP* | Japan | 1314 | 3.6 | 4.2 | 4.3 | 3.4 | **15.9** | **18.4** | **11.3** | **13.7** |
| MX | Mexico | 960 | 2.0 | 0.9 | 0.9 | 0.9 | 5.2 | 4.9 | 2.1 | 2.8 |
| NL* | Netherlands | 1267 | 2.6 | 1.2 | 1.0 | 0.9 | **10.3** | **12.5** | 4.1 | 4.9 |
| NZ* | New Zealand | 1069 | 0.7 | 1.1 | 1.6 | 1.3 | 5.1 | 6.5 | 3.2 | 3.6 |
| NO* | Norway | 1518 | **25.0** | 2.1 | 2.5 | 1.5 | 7.9 | 9.7 | 5.5 | 9.4 |
| PH | Philippines | 1207 | 1.7 | 1.6 | 1.9 | 1.4 | 3.0 | 3.3 | 0.7 | 0.7 |
| PL | Poland | 726 | 2.3 | 0.6 | 1.0 | 1.0 | 9.5 | **10.7** | 7.3 | 8.7 |
| RU | Russia | 1467 | 1.0 | 0.9 | 0.6 | 1.2 | **11.0** | **14.7** | 6.1 | 5.8 |
| SK* | Slovakia | 985 | 3.9 | 1.9 | 2.1 | 2.3 | 8.2 | 9.2 | 3.6 | 3.8 |
| SI* | Slovenia | 978 | 1.9 | 1.8 | 2.0 | 1.7 | 5.7 | 7.9 | 3.2 | 3.3 |
| ZA | South Africa | 2286 | 4.0 | 2.3 | 2.4 | 2.0 | 9.4 | **10.1** | 7.9 | 8.3 |
| SR | Suriname | 1346 | **12.9** | 5.9 | 6.5 | 7.6 | **16.7** | **16.8** | **12.5** | **11.7** |
| CH* | Switzerland | 3270 | 1.0 | 0.8 | 0.9 | 0.4 | 5.3 | 9.3 | 3.5 | 4.9 |
| TH | Thailand | 1110 | 1.3 | 1.1 | 1.9 | 1.8 | 6.2 | 8.5 | **10.4** | **11.0** |
| US | United States | 571 | 1.6 | 3.7 | 3.7 | 3.7 | 7.0 | 6.5 | 4.7 | 6.5 |
| US* | United States | 557 | 1.3 | 2.2 | 2.5 | 2.2 | 5.7 | 5.9 | 2.9 | 4.3 |
| Total | | 40226 |  | | | | | | | |

Notes: *** countries with an asterisk used (partly) self-completion modes including arriving with the interviewer, mailed to the respondent, CASI, and web questionnaire. Countries with a rate of missing values exceeding 10% for the given variable are highlighted in bold.

**Table S2** Psychometric properties of the scales – GOHIE scale (General Online Health Information Engagement)

| **Code** | **Country** | **N** | **Principal Component Analysis (PCA)** | | **Component Matrix – Component 1** | | | | **Component Matrix – Component 2** | | | | **Categorical omega [95% CI]** |
| --- | --- | --- | --- | --- | --- | --- | --- | --- | --- | --- | --- | --- | --- |
|  |  |  | KMO | Bartlett’s test of sphericity | V1 | V2 | V3 | V4 | V1 | V2 | V3 | V4 |  |
| AU* | Australia | 1010 | 0.760 | <0.001 | 0.742 | 0.835 | 0.794 | 0.685 |  |  |  |  | 0.890 [0.864;0.920] |
| AT* | Austria | 1502 | 0.704 | <0.001 | 0.682 | 0.807 | 0.768 | 0.555 |  |  |  |  | 0.834 [0.812;0.855] |
| CN | China | 1833 | 0.764 | <0.001 | 0.755 | 0.829 | 0.745 | 0.750 |  |  |  |  | 0.868 [0.843;0.893] |
| TW | Taiwan | 1389 | 0.749 | <0.001 | 0.786 | 0.862 | 0.714 | 0.710 |  |  |  |  | 0.869 [0.836;0.907] |
| HR | Croatia | 1068 | 0.775 | <0.001 | 0.711 | 0.827 | 0.749 | 0.812 |  |  |  |  | 0.885 [0.859;0.910] |
| CZ | Czech Republic | 1148 | 0.738 | <0.001 | 0.692 | 0.769 | 0.721 | 0.702 |  |  |  |  | 0.858 [0.831;0.878] |
| DK* | Denmark | 1666 | 0.689 | <0.001 | 0.652 | 0.768 | 0.728 | 0.598 |  |  |  |  | 0.790 [0.760;0.815] |
| FI* | Finland | 982 | 0.725 | <0.001 | 0.762 | 0.742 | 0.749 | 0.610 |  |  |  |  | 0.782 [0.745;0.816] |
| FR | France | 1584 | 0.718 | <0.001 | 0.656 | 0.814 | 0.776 | 0.637 |  |  |  |  | 0.782 [0.744;0.816] |
| DE* | Germany | 1611 | 0.741 | <0.001 | 0.729 | 0.809 | 0.734 | 0.681 |  |  |  |  | 0.782 [0.743;0.815] |
| HU | Hungary | 942 | 0.749 | <0.001 | 0.692 | 0.864 | 0.711 | 0.812 |  |  |  |  | 0.867 [0.837;0.889] |
| IS* | Iceland | 1083 | 0.689 | <0.001 | 0.687 | 0.764 | 0.781 | 0.504 |  |  |  |  | 0.763 [0.727;0.799] |
| IN | India | 1533 | 0.658 | <0.001 | 0.592 | 0.737 | 0.740 | 0.708 |  |  |  |  | 0.100 [0.0008;0.430] |
| IL* | Israel | 1183 | 0.719 | <0.001 | 0.694 | 0.731 | 0.729 | 0.627 |  |  |  |  | 0.759 [0.718;0.792] |
| IT | Italy | 1061 | 0.737 | <0.001 | 0.509 | 0.846 | 0.809 | 0.795 |  |  |  |  | 0.881 [0.854;0.908] |
| JP* | Japan | 1314 | 0.786 | <0.001 | 0.798 | 0.831 | 0.808 | 0.709 |  |  |  |  | 0.848 [0.823;0.872] |
| MX | Mexico | 960 | 0.652 | <0.001 | 0.507 | 0.788 | 0.813 | 0.582 |  |  |  |  | 0.745 [0.670;0.804] |
| NL* | Netherlands | 1267 | 0.747 | <0.001 | 0.739 | 0.804 | 0.772 | 0.665 |  |  |  |  | 0.845 [0.818;0.872] |
| NZ* | New Zealand | 1069 | 0.729 | <0.001 | 0.699 | 0.869 | 0.823 | 0.715 |  |  |  |  | 0.903 [0.872;0.931] |
| NO* | Norway | 1518 | 0.650 | <0.001 | 0.598 | 0.746 | 0.699 | 0.541 |  |  |  |  | 0.691 [0.641;0.735] |
| PH | Philippines | 1207 | 0.761 | <0.001 | 0.631 | 0.846 | 0.798 | 0.790 |  |  |  |  | 0.887 [0.857;0.925] |
| PL | Poland | 726 | 0.636 | <0.001 | 0.001 | 0.782 | 0.774 | 0.721 | 0.988 | 0.009 | 0.132 | -0.135 | 0.708 [0.655;0.759] |
| RU | Russia | 1467 | 0.753 | <0.001 | 0.711 | 0.796 | 0.797 | 0.673 |  |  |  |  | 0.892 [0.871;0.913] |
| SK* | Slovakia | 985 | 0.693 | <0.001 | 0.478 | 0.818 | 0.717 | 0.788 |  |  |  |  | 0.845 [0.798;0.889] |
| SI* | Slovenia | 978 | 0.680 | <0.001 | 0.585 | 0.775 | 0.763 | 0.641 |  |  |  |  | 0.785 [0.740;0.824] |
| ZA | South Africa | 2286 | 0.770 | <0.001 | 0.617 | 0.850 | 0.846 | 0.809 |  |  |  |  | 0.912 [0.891;0.936] |
| SR | Suriname | 1346 | 0.746 | <0.001 | 0.584 | 0.843 | 0.833 | 0.760 |  |  |  |  | 0.833 [0.798;0.871] |
| CH* | Switzerland | 3270 | 0.736 | <0.001 | 0.748 | 0.777 | 0.737 | 0.640 |  |  |  |  | 0.825 [0.809;0.840] |
| TH | Thailand | 1110 | 0.680 | <0.001 | 0.374 | 0.889 | 0.849 | 0.770 |  |  |  |  | 0.873 [0.839;0.911] |
| US | United States | 571 | 0.755 | <0.001 | 0.703 | 0.793 | 0.755 | 0.719 |  |  |  |  | 0.803 [0.753;0.845] |
| US* | United States | 557 | 0.666 | <0.001 | 0.539 | 0.820 | 0.749 | 0.636 |  |  |  |  | 0.743 [0.687;0.792] |

Notes: *** countries with an asterisk used (partly) self-completion modes including arriving with the interviewer, mailed to the respondent, CASI, and web questionnaire

**Table S3** Psychometric properties of the scales – DHUS scale (Digital Health Usefulness Scale)

| **Code** | **Country** | **N** | **Principal Component Analysis (PCA)** | | **Component Matrix – Component 1** | | | | **Component Matrix – Component 2** | | | | **Categorical omega [95% CI]** |
| --- | --- | --- | --- | --- | --- | --- | --- | --- | --- | --- | --- | --- | --- |
|  |  |  | KMO | Bartlett’s test of sphericity | V5 | V6 | V7 | V8 | V5 | V6 | V7 | V8 |  |
| AU* | Australia | 1010 | 0.648 | <0.001 | 0.679 | 0.723 | 0.747 | 0.782 |  |  |  |  | 0.892 [0.865;0.918] |
| AT* | Austria | 1502 | 0.682 | <0.001 | 0.680 | 0.734 | 0.772 | 0.769 |  |  |  |  | 0.831 [0.812;0.854] |
| CN | China | 1833 | 0.663 | <0.001 | 0.668 | 0.753 | 0.751 | 0.750 |  |  |  |  | 0.865 [0.844;0.894] |
| TW | Taiwan | 1389 | 0.658 | <0.001 | 0.739 | 0.761 | 0.676 | 0.678 |  |  |  |  | 0.871 [0.837;0.905] |
| HR | Croatia | 1068 | 0.691 | <0.001 | 0.791 | 0.790 | 0.759 | 0.775 |  |  |  |  | 0.883 [0.860;0.910] |
| CZ | Czech Republic | 1148 | 0.672 | <0.001 | 0.745 | 0.790 | 0.777 | 0.779 |  |  |  |  | 0.855 [0.833;0.878] |
| DK* | Denmark | 1666 | 0.634 | <0.001 | 0.675 | 0.733 | 0.720 | 0.765 |  |  |  |  | 0.793 [0.762;0.817] |
| FI* | Finland | 982 | 0.651 | <0.001 | 0.674 | 0.758 | 0.706 | 0.709 |  |  |  |  | 0.785 [0.744;0.816] |
| FR | France | 1584 | 0.657 | <0.001 | 0.733 | 0.719 | 0.754 | 0.773 |  |  |  |  | 0.786 [0.746;0.818] |
| DE* | Germany | 1611 | 0.645 | <0.001 | 0.695 | 0.727 | 0.722 | 0.739 |  |  |  |  | 0.826 [0.800;0.848] |
| HU | Hungary | 942 | 0.731 | <0.001 | 0.789 | 0.848 | 0.827 | 0.841 |  |  |  |  | 0.865 [0.840;0.889] |
| IS* | Iceland | 1083 | 0.570 | <0.001 | 0.057 | 0.146 | 0.866 | 0.857 | 0.849 | 0.824 | 0.080 | 0.127 | 0.761 [0.727;0.798] |
| IN | India | 1533 | 0.587 | <0.001 | 0.647 | 0.659 | 0.593 | 0.627 |  |  |  |  | 0.215 [0.0009;0.426] |
| IL* | Israel | 1183 | 0.653 | <0.001 | 0.649 | 0.712 | 0.727 | 0.762 |  |  |  |  | 0.763 [0.717;0.791] |
| IT | Italy | 1061 | 0.676 | <0.001 | 0.796 | 0.807 | 0.798 | 0.820 |  |  |  |  | 0.877 [0.854;0.908] |
| JP* | Japan | 1314 | 0.704 | <0.001 | 0.746 | 0.785 | 0.720 | 0.771 |  |  |  |  | 0.844 [0.823;0.871] |
| MX | Mexico | 960 | 0.667 | <0.001 | 0.681 | 0.726 | 0.792 | 0.821 |  |  |  |  | 0.752 [0.667;0.802] |
| NL* | Netherlands | 1267 | 0.595 | <0.001 | 0.869 | 0.861 | 0.128 | 0.151 | 0.126 | 0.154 | 0.863 | 0.857 | 0.849 [0.819;0.870] |
| NZ* | New Zealand | 1069 | 0.645 | <0.001 | 0.646 | 0.689 | 0.736 | 0.782 |  |  |  |  | 0.915 [0.873;0.932] |
| NO* | Norway | 1518 | 0.597 | <0.001 | 0.087 | 0.154 | 0.882 | 0.847 | 0.835 | 0.815 | 0.064 | 0.194 | 0.697 [0.639;0.731] |
| PH | Philippines | 1207 | 0.645 | <0.001 | 0.558 | 0.755 | 0.790 | 0.780 |  |  |  |  | 0.882 [0.855;0.922] |
| PL | Poland | 726 | 0.650 | <0.001 | 0.741 | 0.730 | 0.747 | 0.699 |  |  |  |  | 0.713 [0.651;0.756] |
| RU | Russia | 1467 | 0.693 | <0.001 | 0.769 | 0.787 | 0.772 | 0.761 |  |  |  |  | 0.898 [0.870;0.912] |
| SK* | Slovakia | 985 | 0.619 | <0.001 | 0.926 | 0.900 | 0.210 | 0.156 | 0.143 | 0.235 | 0.884 | 0.900 | 0.852 [0.796;0.888] |
| SI* | Slovenia | 978 | 0.664 | <0.001 | 0.692 | 0.746 | 0.789 | 0.765 |  |  |  |  | 0.791 [0.740;0.824] |
| ZA | South Africa | 2286 | 0.746 | <0.001 | 0.696 | 0.800 | 0.794 | 0.785 |  |  |  |  | 0.908 [0.891;0.935] |
| SR | Suriname | 1346 | 0.629 | <0.001 | 0.656 | 0.704 | 0.725 | 0.736 |  |  |  |  | 0.836 [0.798;0.871] |
| CH* | Switzerland | 3270 | 0.677 | <0.001 | 0.667 | 0.748 | 0.733 | 0.758 |  |  |  |  | 0.821 [0.809;0.840] |
| TH | Thailand | 1110 | 0.687 | <0.001 | 0.784 | 0.789 | 0.788 | 0.822 |  |  |  |  | 0.868 [0.838;0.914] |
| US | United States | 571 | 0.651 | <0.001 | 0.658 | 0.679 | 0.754 | 0.775 |  |  |  |  | 0.815 [0.751;0.846] |
| US* | United States | 557 | 0.665 | <0.001 | 0.606 | 0.632 | 0.752 | 0.744 |  |  |  |  | 0.754 [0.690;0.793] |

Notes: *** countries with an asterisk used (partly) self-completion modes including arriving with the interviewer, mailed to the respondent, CASI, and web questionnaire

**Table S4** BAMI Global Fit Indices for GOHIE-INT and GOHIE-SELF (General Online Health Information Engagement) and DHUS-INT and DHUS-SELF (Digital Health Usefulness Scale)

| **Scale** | | **Sample size** | **Prior variance** | **PPP** | **95% CI** | **BRMSEA** | **BCFI** | **BTLI** | **BIC** | **DIC** |
| --- | --- | --- | --- | --- | --- | --- | --- | --- | --- | --- |
| **GOHIE** | GOHIE-INT | 13 groups, N=15,186 | 0.0001 | 0.000 | [3265.581;3399.139] | 0.173 | 0.766 | 0.809 | 185469.439 | 183910.349 |
|  |  |  | 0.001 | 0.000 | [1608.106;1801.665] | 0.137 | 0.880 | 0.879 | 183823.800 | 182300.583 |
|  |  |  | 0.005 | 0.000 | [409.380;560.637] | 0.089 | 0.966 | 0.949 | 182576.438 | 181105.932 |
|  |  |  | 0.010 | 0.000 | [166.674;298.652] | 0.067 | 0.984 | 0.971 | 182314.031 | 180861.528 |
|  |  |  | 0.025 | 0.001 | [27.086;140.022] | 0.040 | 0.994 | 0.990 | 182156.961 | 180714.564 |
|  |  |  | **0.05** | **0.033** | **[-3.071;100.881]** | **0.026** | **0.997** | **0.996** | **182120.808** | **180662.811** |
|  | GOHIE-SELF | 13 groups, N=15,843 | 0.0001 | 0.000 | [3814.807;3961.137] | 0.183 | 0.614 | 0.684 | 189290.478 | 187723.605 |
|  |  |  | 0.001 | 0.000 | [1587.383;1852.560] | 0.134 | 0.830 | 0.829 | 187082.019 | 185570.415 |
|  |  |  | 0.005 | 0.000 | [326.410;479.392] | 0.082 | 0.960 | 0.936 | 185754.268 | 184284.768 |
|  |  |  | 0.010 | 0.000 | [115.687;244.617] | 0.058 | 0.983 | 0.969 | 185524.655 | 184067.517 |
|  |  |  | 0.025 | 0.006 | [17.145;124.405] | 0.032 | 0.993 | 0.990 | 185408.716 | 183949.891 |
|  |  |  | **0.05** | **0.035** | **[-3.803;99.259]** | **0.018** | **0.996** | **0.997** | **185383.770** | **183873.933** |
| **DHUS** | DHUS-INT | 15 groups, N=17,970 | 0.0001 | Non-convergent | | | | | | |
|  |  |  | 0.001 | 0.000 | [830.440;983.986] | 0.025 | 0.949 | 0.996 | 182095.844 | 179090.196 |
|  |  |  | 0.005 | 0.000 | [134.080;268.508] | 0.062 | 0.989 | 0.976 | 181375.744 | 179535.809 |
|  |  |  | 0.010 | 0.000 | [47.037;166.585] | 0.045 | 0.995 | 0.988 | 181274.210 | 179443.949 |
|  |  |  | 0.025 | 0.012 | [7.544;120.207] | 0.024 | 0.997 | 0.997 | 181225.435 | 179366.395 |
|  |  |  | **0.05** | **0.031** | **[-3.132;111.854]** | **0.014** | **0.998** | **0.999** | **181215.644** | **179253.100** |
|  | DHUS-SELF | 16 groups, N=21,182 | 0.0001 | 0.000 | [1478.749;1612.886] | 0.107 | 0.903 | 0.908 | 216614.670 | 214491.188 |
|  |  |  | 0.001 | 0.000 | [473.732;627.913] | 0.076 | 0.966 | 0.954 | 215586.308 | 213526.299 |
|  |  |  | 0.005 | 0.000 | [59.392;184.246] | 0.042 | 0.993 | 0.986 | 215129.820 | 213122.918 |
|  |  |  | 0.010 | 0.018 | [5.432;121.299] | 0.029 | 0.997 | 0.993 | 215063.329 | 213067.227 |
|  |  |  | **0.025** | **0.089** | **[-16.692;95.135]** | **0.016** | **0.999** | **0.998** | **215032.683** | **213017.353** |
|  |  |  | 0.05 | 0.107 | [-21.032;91.935] | 0.009 | 0.999 | 0.999 | 215026.761 | 212893.308 |

Notes: Acceptable model fit was defined by a 95% CI including zero, BRMSEA < 0.06, BCFI and BTLI > 0.95, and improvements of ≥20 in BIC or ≥14 in DIC compared with the preceding model. The final models demonstrating good fit and acceptably low prior variances are highlighted in bold.

**Table S5** Countries with significant deviations of item loadings and intercepts relative to the average across all countries for GOHIE (General Online Health Information Engagement) and DHUS scales (Digital Health Usefulness Scale)

| Scales | Data collection mode/Item | „During the past 12 months, how often, if at all, did you use the internet on any device (such as computers, tablets and smartphones) to look for health or medical information for yourself or someone else?“  (6-point verbal scale) | "During the past 12 months, how often, if at all, have you used the internet to look for information on the following topics?: information on healthy lifestyle."  (5-point verbal scale) | "During the past 12 months, how often, if at all, have you used the internet to look for information on the following topics?: information related to anxiety, stress, or similar problems."  (5-point verbal scale) | "During the past 12 months, how often, if at all, have you used the internet to look for information on the following topics?: information on vaccinations."  (5-point verbal scale) |
| --- | --- | --- | --- | --- | --- |
|  |  | **v1** | **v2** | **v3** | **v4** |
| **GOHIE** | INT | **Taiwan**, **Hungary**, Italy, **Mexico**, Philippines, Poland, Russia, South Africa, **Suriname**, Thailand, **United States** | **Hungary**, Philippines, **South Africa**, Thailand, **United States** | **Taiwan**, **Hungary**, **Mexico**, Poland, Russia, **South Africa**, **Suriname**, **United States** | **Taiwan**, Czech Republic, **Mexico**, **South Africa**, **Suriname** |
|  | SELD | Australia, **Finland**, France, Iceland, **Israel**, Norway, **Slovakia** | **Finland**, **Israel**, Japan, **Slovakia** | Australia, **Finland**, **Slovakia** | Austria, Denmark, **Finland**, Germany, Iceland, **Israel**, Japan, Norway, **Slovakia** |
|  |  | „During the past 12 months, information on the internet affected my health behaviour in a positive way.“  (5-point verbal scale) | „During the past 12 months, information on the internet helped me understand what a doctor tried to explain to me.“ (5-point verbal scale) | „The internet is useful to help people decide if their symptoms are serious enough to go to the doctor.“  (5-point verbal scale) | „The internet is useful to check that the doctor is giving people appropriate advice.“  (5-point verbal scale) |
|  |  | **v5** | **v6** | **v7** | **v8** |
| **DHUS** | INT | **Philippines**, United States | **India**, **Italy** | Taiwan, **India**, **Italy**, Mexico, **Philippines**, Russia, South Africa, Suriname, United States | Czech Republic, **India**, **Italy**, **Philippines**, Poland, Russia, Suriname |
|  | SELF | Finland, United States | **France**, **Iceland**, Slovakia | Australia, **France**, **Iceland**, Israel, Japan, Netherlands, Norway | Finland, **France**, Germany, **Iceland**, Japan, Slovakia, Switzerland |

Notes: countries in bold are those with more frequent deviated item parameters

**Fig. S1** Approximate Measurement Non-Invariance by Country and Item (parameters NU/intercepts) - Deviation Heatmap: General Online Health Information Engagement (GOHIE scale)

Notes: Asterisks indicate intercepts significantly deviating from the cross-national average (non-invariant parameters). Darker color intensity represents greater deviation from the average estimate. Boldly outlined cells mark items where both the item intercept and loading diverged significantly, indicating violations of metric and scalar approximate invariance.

**Fig. S2** Approximate Measurement Non-Invariance by Country and Item (parameters NU/intercepts) - Deviation Heatmap: Digital Health Usefulness Scale (DHUS scale)

Notes: Asterisks indicate intercepts significantly deviating from the cross-national average (non-invariant parameters). Darker color intensity represents greater deviation from the average estimate. Boldly outlined cells mark items where both the item intercept and loading diverged significantly, indicating violations of metric and scalar approximate invariance.
